# GENDER-SPECIFIC ASSOCIATION BETWEEN DEPRESSION AND MORTALITY IN KOREAN OLDER ADULTS: A LONGITUDINAL STUDY

**DOI:** 10.1093/geroni/igad104.2871

**Published:** 2023-12-21

**Authors:** Jungwon Cho, Jae Jun Lee, Eunhee Cho

**Affiliations:** Yonsei University College of Nursing, Seoul, Republic of Korea; Yonsei University College of Nursing, Seoul, Republic of Korea; Yonsei University College of Nursing, Seoul, Republic of Korea

## Abstract

Late-life depression has become a significant public health problem because it adversely affects physical health, social function, and quality of life. Therefore, prevention and treatment of depression are necessary. Previous studies have indicated that depression in older adults is associated with mortality. However, research on mortality according to gender differences has not been actively conducted. Thus, this study aims to investigate gender differences between depression and mortality among Korean older adults. The data were collected from the Korean Longitudinal Study of Aging (KLoSA), which performed a nationwide aging panel survey for adults aged over 45 years between 2006 and 2020. Out of 10,254 participants, this study included 3,689 participants who were aged 65 or older and assessed depression through CESD-10. The Cox proportional-hazards model was used to calculate Hazard Ratios (HR) for mortality stratified by gender. Of the 1,599 male and 2,090 female older adults, depression was significantly higher in females (16.7%) than in males (8.8%). However, the effect of depression on mortality was significant only in males (male: HR = 1.50, 95% CI = 1.19–1.89; female: HR = 1.10, 95% CI = 0.93–1.31). Depression is more common in females, whereas the effect of depression on mortality was only significant in males. This suggests that health providers need to prevent and care for depression to decrease mortality, especially in males. Further studies are needed to explore the reasons for gender differences between depression and mortality and develop interventions to manage depression in older adults.

